# *Stanniocalcin2*, but Not *Stanniocalcin1*, Responds to Hypoxia in a HIF1-Dependent Manner in the Retina

**DOI:** 10.3389/fnins.2022.882559

**Published:** 2022-06-23

**Authors:** Divya Ail, Marijana Samardzija, Andy C. M. Chang, Jadwiga Keck, Roger R. Reddel, Christian Grimm

**Affiliations:** ^1^Sorbonne Université, INSERM, CNRS, Institut de la Vision, Paris, France; ^2^Lab for Retinal Cell Biology, Department of Ophthalmology, University Hospital Zurich, University of Zurich, Zurich, Switzerland; ^3^Neuroscience Center Zurich (ZNZ), University of Zurich, Zurich, Switzerland; ^4^Faculty of Medicine and Health, Children’s Medical Research Institute, University of Sydney, Westmead, NSW, Australia; ^5^Zurich Center for Integrative Human Physiology (ZIHP), University of Zurich, Zurich, Switzerland

**Keywords:** *stanniocalcin1 (Stc1)*, *stanniocalcin2 (Stc2)*, hypoxia, hypoxia-inducible factor 1a (HIF1a), retinal stress

## Abstract

The quest for neuroprotective factors that can prevent or slow down the progression of retinal degeneration is still ongoing. Acute hypoxic stress has been shown to provide transient protection against subsequent damage in the retina. Stanniocalcins – STC1 and STC2 – are secreted glycoproteins that are hypoxia-regulated and were shown to be cytoprotective in various *in vitro* studies. Hence, we investigated the expression of stanniocalcins in the normal, degenerating and hypoxic retina. We show that the expression of *Stc1* and *Stc2* in the retina was detectable as early as postnatal day 10 and persisted during aging. Retinal expression of *Stc2*, but not *Stc1*, was induced in mice in an *in vivo* model of acute hypoxia and a genetic model of chronic hypoxia. Furthermore, we show that HIF1, not HIF2, is responsible for regulating *Stc2* in cells with the molecular response to hypoxia activated due to the absence of von Hippel Lindau protein. Surprisingly, *Stc2* was not normally expressed in photoreceptors but in the inner retina, as shown by laser capture microdissection and immunofluorescence data. The expression of both *Stc1* and *Stc2* remained unchanged in the degenerative retina with an almost complete loss of photoreceptors, confirming their expression in the inner retina. However, the absence of either *Stc1* or *Stc2* had no effect on retinal architecture, as was evident from retinal morphology of the respective knockout mice. Taken together our data provides evidence for the differential regulation of STC1 and STC2 in the retina and the prospect of investigating STC2 as a retinal neuroprotective factor.

## Introduction

Retinal degenerative diseases not only reduce the quality of life of the patients but are also a huge economic burden. Strategies that focus on supplying neuroprotective factors as treatment for these diseases are promising. Hence, there is an immense interest in identification and characterization of such neuroprotective factors that can promote survival of retinal neurons and slow down or prevent the progression of disease (Pardue and Allen, 2018). Subjecting the retinal tissue to acute stress such as hypoxic stress can result in the secretion of such neuroprotective factors (Thiersch et al., 2008).

The corpuscle of Stannius (named after Hermann Friedrich Stannius who discovered it in 1839 in bony fish) secrets a glycoprotein hormone named stanniocalcin (STC) (reviewed in Chang et al., 2003). The human *STC1* gene was first identified in a screen for genes involved in the control of proliferation and almost 60% of the amino acids it encodes are identical to fish STC (Chang et al., 1995; Olsen et al., 1996). *STC2* was identified later (Chang and Reddel, 1998; DiMattia et al., 1998; Ishibashi et al., 1998) and shares only 30% nucleotide identity with *STC1* suggesting a common ancestor (Wagner and DiMattia, 2006). Both STC1 and STC2 have signal peptides of about 24 amino acids suggesting that they are secreted proteins (Moore et al., 1999).

Both stanniocalcins have been implicated in cancer formation (Yeung et al., 2012; Joshi, 2020). The tumor microenvironment is often hypoxic (Vaupel et al., 1989), and several studies have shown *Stc1* and *Stc2* to be regulated by hypoxia. Increased expression of *Stc1* was reported in mice that were preconditioned by hypoxia resulting in ischemic tolerance in the heart (Westberg et al., 2007a) and brain (Westberg et al., 2007b). Additionally, hypoxic exposure of various cancer cell lines resulted in the upregulation of *STC1* expression (Yeung et al., 2005). Similarly, STC2 was induced by hypoxia in proximal tubular epithelial cells (Leonard et al., 2003). Analysis of promoters of both *STC1* and *STC2* revealed putative hypoxia responsive elements (HRE) and *in vitro* studies using mutated versions of these sites suggested that both *STC1* and *STC2* are HIF1 (hypoxia inducible factor 1) target genes, although the association for *STC1* was not consistent (Law et al., 2008, 2010; Law and Wong, 2010b).

A few studies have investigated the molecular mechanisms and subcellular functions of the stanniocalcins, with several focusing on their role in calcium homeostasis (Zeiger et al., 2011; López et al., 2018). Other studies reported the identification of STC1 binding sites on the mitochondrial membrane (McCudden et al., 2002) or an uncoupling effect of STC1 on oxidative phosphorylation, suggesting a role in intracellular metabolism (Ellard et al., 2007), despite the presence of a signal peptide for secretion. Using fibroblasts from *Stc2* knockout mice, Zeiger et al. (2011) showed that STC2 is a negative regulator of store-operated calcium entry and several cell culture studies have reported cytoprotective effects of STC2 on mesenchymal cells (Kim et al., 2015), hepatocytes (Joshi et al., 2015), and neuronal cells (Zhang et al., 2000; Ito et al., 2004). Studies in mice have shown *in vivo* protective effects of STC2 on pancreas (Fazio et al., 2011), liver (Zhao J. et al., 2018), and adipose tissue (Sarapio et al., 2019).

Not much is known about the function of stanniocalcins in the retina. However, STC1 increased cell viability of the retinal cell line RGC-5 (Kim et al., 2013) and rescued retinal degeneration in two rat models after intravitreal protein injection or AAV-mediated gene delivery (Roddy et al., 2012, 2017). STC1 was protective in a model of retinopathy of prematurity (Dalvin et al., 2020), and was implicated in the development of choroidal neovascularization (Zhao M. et al., 2018), glaucoma pathogenesis (Lu et al., 2019), and the protection of ganglion cells (Kim et al., 2013). No studies report on the role of STC2 in the retina so far. As the retina is a tissue with a high oxygen demand, it is very sensitive to variations in oxygen levels and hypoxia has been implicated in degenerative diseases of the retina (Arjamaa and Nikinmaa, 2006; Grimm and Willmann, 2012; Lange and Bainbridge, 2012). Here we report for the first time on differences between the hypoxic regulation of *Stc1* and *Stc2* in the mouse retina.

## Materials and Methods

### Animals and Hypoxic Exposure

Animals were treated in accordance with the regulations of the veterinary authority of Zurich and the statement of the Association for Research in Vision and Ophthalmology (ARVO) for the use of animals in vision research. All mice were kept at the Laboratory Animal Services Center (LASC) of the University of Zurich in a light-dark (12 h:12 h) cycle with food and water *ad libitum*. Light was maintained at 60 lux at cage level during the light period. Mice were euthanized by CO_2_ inhalation followed by cervical dislocation. C57BL/6 mice were purchased from Charles River (Chatillon-sur-Chalaronne, France), 129S6 mice from Taconic (Ejby, Denmark), *rd10* mice (C57BL/6 background) from Jackson Laboratories (Bar Harbor, ME, United States) and Balb/c mice were offspring from an in-house breeding. The master regulator of the hypoxic response is the hypoxia-inducible factor 1 (HIF1) belonging to a family of proteins that also includes HIF2 and HIF3 (Semenza, 2012). HIF1 is a heterodimer composed of a nuclear-localized beta subunit and an oxygen labile alpha subunit (Wang et al., 1995) that is constitutively produced but undergoes immediate degradation in normoxia *via* an oxygen-dependent mechanism (Maxwell et al., 1999). Since degradation requires VHL (von Hippel Lindau protein), the absence of functional VHL leads to a stabilization of HIFA subunits (Jaakkola et al., 2001), the activation of HIF transcription factors and induction of the hypoxic response even in normoxic cells (Krieg et al., 2000; Lange et al., 2011). The mice lacking the VHL in the rod photoreceptors serve as a model for chronic hypoxia-like response in the retina. *Opsin-cre;Vhl*^flox/flox^** (*rod^Δ^*^Vhl^**), *opsin-cre;Vhl*^flox/flox^*;Hif1a*^flox/flox^** (*rod^Δ^*^Vhl;Hif^*^1^*^a^**), *opsin-cre;Vhl*^flox/flox^*;Hif2a*^flox/flox^** (*rod^Δ^*^Vhl;Hif^*^2^*^a^**) and *opsin-cre;Vhl*^flox/flox^*;Hif1*^flox/flox^*;Hif2a*^flox/flox^** (*rod^Δ^*^Vhl;Hif^*^1^*^a;Hif^*^2^*^a^**) mice were generated and genotyped as described (Lange et al., 2011; Barben et al., 2018). Mice with floxed genes but without Opsin-cre served as controls. The STC1-/- and STC2-/- mice were generated as described earlier (Chang et al., 2005, 2008). 129S6 and Balb/C wild type mice were exposed to hypoxia in a hypoxia chamber by reducing O_2_ levels by 2% every 10 min until a level of 7% O_2_ was reached. Hypoxia was maintained for 6 h before mice were either euthanized (immediate time-point) or reoxygenated in normal room air (up to 16 h) before euthanasia and tissue collection (Grimm et al., 2002). Since the genetic background of the mouse strain does not influence their response to hypoxia, different strains of WT mice were used for hypoxia experiments as per availability at various time and age-points. Balb/c mice were used for hypoxia and reoxygenation experiment. C57BL/6 mice at 7 weeks and 7 months of age were used for testing gene expression post-hypoxia at different ages. Gene expression in *rd10* mice was compared to C57BL/6 WT animals and the strain used for laser capture microdissection experiment was 129S6.

### Morphology and Light Microscopy

Eyes were enucleated and fixed in 2.5% glutaraldehyde in 0.1 M cacodylate buffer (pH 7.3) at 4°C overnight. After fixation, cornea and lens were removed, and the eyecup was separated into a superior and an inferior half by cutting through the optic nerve head. The trimmed tissue was washed in cacodylate buffer, contrasted with osmium tetroxide (1%) for 1 h at room temperature, dehydrated by incubations in increasing ethanol concentrations, and embedded in Epon 812. Semi-thin sections (0.5 μm) were prepared and counterstained with toluidine blue. An Axioplan digitalized microscope (Zeiss Meditec, Jena, Germany) was used to examine the slides. Shown are representative images of *N* = 3 independent animals per strain.

### RNA Isolation, cDNA Synthesis, and Semiquantitative Real-Time PCR

Retinas were isolated through a corneal incision and immediately snap frozen in liquid nitrogen. Total RNA was isolated using the RNeasy isolation kit (catalog number: 74104, Qiagen, Hilden, Germany) or the High Pure RNA isolation kit (catalog number: 11828665001, Roche Diagonistics, Basel, Switzerland). Residual genomic DNA was removed by a DNase I incubation step. Total RNA (650–1000 ng) was reverse transcribed using oligo(dT) and M-MLV reverse transcriptase (catalog number: M1701, Promega, Madison, WI, United States). 10 ng of cDNA was used for gene expression analysis by semiquantitative real-time PCR using a LightCycler 480 instrument (Roche Diagnostics), the LightCycler 480 SYBR Green I Master mix (catalog number: 04887352001, Roche Diagonistics), and specific primer pairs (Table 1). The primer pairs were designed to span large intronic sequences or to cover exon-intron boundaries. Gene expression was normalized to ß-actin (*Actb*), and relative quantification was calculated using the comparative threshold method (ΔΔC_T_).

**TABLE 1:** Primers used for semiquantitative real-time PCR.

Gene	Forward primer (5′-3′)	Reverse primer (5′-3′)	Annealing temp (°C)	Amplicon size (bp)
*Actb*	CAACGGCTCCGGCATGTGC	CTCTTGCTCTGGGCCTCG	62	153
*Adm*	TTCGCAGTTCCGAAAGAAGT	GGTAGCTGCTGGATGCTTGTA	62	77
*Epo*	GCCCTGCTAGCCAATTCC	GGCGACATCAATTCCTTCTG	60	128
*Gnat1*	GAGGATGCTGAGAAGGATGC	TGAATGTTGAGCGTGGTCAT	58	209
*Mct3*	GGCTCAACCCTAAATCCAGA	CTTCGGAGTTTCCTCACCAG	62	75
*Opn4*	CCAGCTTCACAACCAGTCCT	CAGCCTGATGTGCAGATGTC	62	111
*Stc1*	CCGGAAGCCATCACTGAA	GGCTTCGGACAAGTCTGTTG	62	76
*Stc2*	AGCAGGAAGTGTCCAGCAAT	GGTTCACAAGGTCCACATAGG	62	166
*Vsx2*	CCAGAAGACAGGATACAGGTG	GGCTCCATAGAGACCATACT	62	111

### Laser Capture Microdissection

Eyes from 8 weeks old wild type (129S6) mice were enucleated, immediately embedded in tissue freezing medium (Leica Microsystems Nussloch GmbH, Nussloch, Germany), and frozen in a 2-methylbutane bath cooled in liquid nitrogen. 20 μm thick retinal sections were collected on Arcturus PEN Membrane Glass Slides (Applied Biosystems, Foster City, CA, United States). The slides were fixed for 5 min in acetone, followed by 5 min of air drying and two dehydration steps (30 s with 100% ethanol and 5 min with xylol). Retinal layers were isolated using an Arcturus XT Laser Capture Microdissection system (Bucher Biotec AG, Basel, Switzerland) and Arcturus CapSure Macro LCM Caps (Applied Biosystems). Extraction of RNA was done using the Arcturus PicoPure RNA Isolation Kit (Applied Biosystems) according to the manufacturer’s instructions, including a DNase treatment to remove residual genomic DNA. cDNA was synthesized using random hexamer primers (High-Capacity cDNA Reverse Transcription Kit; Applied Biosystems), and gene expression analyzed by semi-quantitative real-time PCR as described in above.

### Immunofluorescence on Retinal Cryosections and Flatmounts

To prepare cryosections, eyes were enucleated and fixed in 4% paraformaldehyde (PFA) overnight. The cornea and lens were removed and the eyecups immersed in 30% sucrose in Phosphate-buffered saline (PBS). Eyecups were embedded in tissue cryoprotective medium (Leica Microsystems Nussloch GmbH, Nussloch, Germany) and frozen in a 2-methylbutane bath cooled with liquid nitrogen. 12 μm thick sections were cut through the optic nerve head with a cryostat (Leica), air-dried, and stored at –80°C until further use. To prepare flatmounts, eyes were enucleated and incubated for 3–5 min in 2% paraformaldehyde prepared in phosphate-buffered saline (PBS). After having removed cornea and lens, the eyeball was cut in a cloverleaf shape by making four incisions. The retina was dissected from the sclera and placed in PBS in a 48-well plate. Dissected retinas were post-fixed at room temperature for 1 h with 4% paraformaldehyde in PBS and washed with PBS. Retinal sections or flatmounts were incubated with blocking solution (3% goat serum/0.3% Triton-X in PBS) for 1 h at room temperature. Primary antibodies were applied and left on sections at 4°C overnight (Table 2). Specificity of the anti-STC2 antibody was tested on Western blots with the antibody detecting a protein of the size of STC2 (34 kDa) but not of STC1 (28 kDa) (not shown). Slides were washed 3 times with PBS followed by incubation with Cy2 or Cy3-conjugated secondary antibodies (Jackson Immunoresearch, Soham, United Kingdom) in blocking solution (dilution = 1:500) for 1 h at room temperature. After 3 PBS washes the sections were stained with DAPI to visualize nuclei and slides mounted with the anti-fade medium Mowiol. Immunofluorescently labeled proteins were imaged using an Axioplan fluorescence microscope (Carl Zeiss AG). Shown are representative images of *N* = 3 independent animals per strain.

**TABLE 2:** Antibodies used for immunofluorescence on retinal sections.

Protein	Host	Supplier	Catalog number	Dilution
BRN3A	Mouse	Chemicon (Millipore); United States	MAB1585	1:300
GFAP	Mouse	Sigma, St. Louis, MO, United States	G3893	1:500
GS	Mouse	Millipore, United States	MAB302	1:500
STC2	Rabbit	Acris antibodies, Herford, Germany	10314-1-AP	1:300
SYN	Mouse	Novocastra, Muttenz, Switzerland	NCL-L-SYNAP-299	1:500

### Statistical Analysis

Statistical analysis was performed using Prism software (GraphPad, San Diego, CA, United States). All data are presented as mean ± standard deviation (SD). The number of samples (N) and the statistical test used for individual experiments are given in the Figure captions. Student’s *t*-tests were used to measure the statistical differences of means and differences between two groups. One-way or two-way ANOVA with respective *post hoc* tests (see Figure captions) were used to compare several groups. *P*-value ≤ 0.05 was considered significant.

## Results

### Expression of *Stc1* and *Stc2* in the Mouse Retina

**FIGURE 1:**
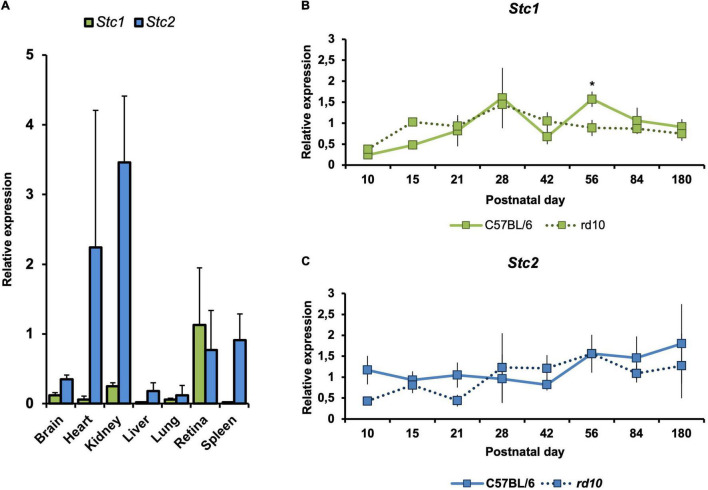
Gene expression of *Stc1* and *Stc2* in the retina during postnatal development, aging and degeneration. **(A)** Relative expression levels of *Stc1* (green bars) and *Stc2* (blue bars) in different tissues isolated from 11 weeks old wild type (C57BL/6) mice. Relative expression levels of *Stc1* **(B)** and *Stc2* **(C)** in the retina of wild type (C57BL/6) mice (solid line) and *rd10* mice (dotted line) from PND10 to PND180. Expression was determined by semi-quantitative real-time PCR and normalized to *Actb* levels. Shown are mean values ± SD of *N* = 3. *P < 0.05. Two-way ANOVA with Sidak’s multiple comparison test.

To investigate *Stc1* and *Stc2* in the retina, we compared their expression levels in the retina to those in other tissues. When normalized to *Actb*, *Stc1* was expressed more strongly in the retina than in any other tissue investigated. *Stc2* was also highly expressed in the retina but did not reach expression levels detected in heart and kidney (Figure 1A).

We also analyzed the expression of *Stc1* and *Stc2* in the retina during postnatal development and aging from postnatal day 10 (PND10) to PND180. Both genes were expressed as early as PND10 in the retinas of wild type mice. While expression of *Stc2* was quite steady during the time frame analyzed, expression of *Stc1* slightly increased during postnatal development and aging (Figures 1B,C – solid lines; period between PND10 and PND28). To investigate whether expression of *Stc1* and *Stc2* is affected by retinal degeneration, we analyzed their mRNA levels in *rd10* mice that are characterized by a progressive loss of rod photoreceptors starting around PND15 and a loss of almost all photoreceptors at 6 months of age (Samardzija et al., 2012). With the exception of PND56 for *Stc1*, expression of *Stc1* and *Stc2* in *rd10* and wild type retinas was comparable at all time points tested (Figures 1B,C – dotted lines), suggesting that these genes may not have a direct or important role in the degenerating retina. Since almost all photoreceptors are lost in *rd10* mice at the late timepoints, the data also suggest that *Stc1* and *Stc2* are not expressed in photoreceptors, or are expressed at very low levels.

### In the Retina *Stc2* but Not *Stc1* Is Responsive to Hypoxia

**FIGURE 2:**
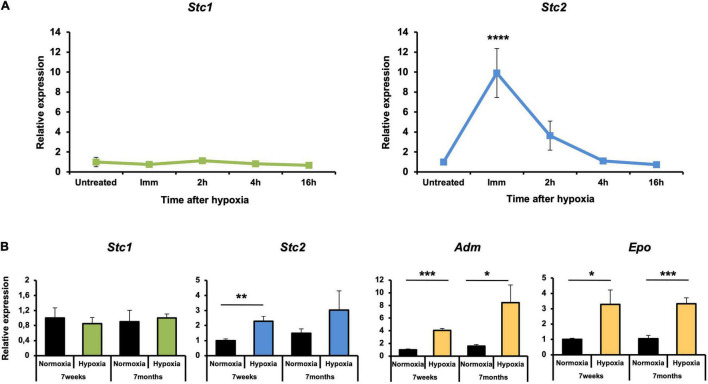
Upregulation of *Stc2* gene expression in response to hypoxia. **(A)** Relative expressions of *Stc1* (left panel) and *Stc2* (right panel) in retinas of 6 weeks old wild type (Balb/c) mice exposed to hypoxia (6 h, 7% O_2_). Tissue was collected immediately after hypoxia (Imm) or after 2, 4, and 16 h of reoxygenation following hypoxia. **(B)** Relative expression of *Stc1*, *Stc2*, *Adm*, and *Epo* in retinas of wild-type (129S6) mice exposed to normoxia (black bars) or hypoxia (tissue collected immediately after 6 h at 7% O_2_) (colored bars) at 7 weeks or 7 months of age. Expression levels were determined by semi-quantitative real-time PCR, normalized to *Actb* and shown relative to the levels in untreated controls (set to 1) **(A)** or to normoxic controls at 7 weeks of age (set to 1) **(B)**. Shown are mean values ± SD of *N* = 3. Significance was tested using one-way ANOVA with Dunnett’s multiple comparison test **(A)** and by Student’s t-test **(B)** (*P < 0.05, **P < 0.01, ***P < 0.001, *P* < 0.0001****).

The retina is a metabolically active tissue with a high oxygen consumption rate (Yu and Cringle, 2001) and therefore is very sensitive to hypoxia (Caprara and Grimm, 2012). Since previous studies have shown STC1 and STC2 to be responsive to hypoxia (see section “Introduction”), we examined *Stc1* and *Stc2* expression in the retina of mice that have been exposed for 6 h to 7% O_2_. Surprisingly, expression of *Stc1* remained unaltered in the hypoxic retina. In contrast, expression of *Stc2* was upregulated ninefold immediately after hypoxia and was still fourfold elevated at 2 h of reoxygenation before decreasing to basal levels after 4 h of reoxygenation (Figure 2A). To test if the hypoxic response was age-related, 7 weeks old and 7 months old mice were exposed to hypoxia. Again, *Stc1* did not respond to the treatment whereas *Stc2* showed significant upregulation in young and a trend toward upregulation in old mice suggesting that the hypoxic induction did not depend on the age of the mice. The known hypoxia-regulated genes *Adm* (adrenomedullin) (Zhao et al., 1996) and *Epo* (erythropoetin) (Grimm et al., 2002) were used as controls and showed an upregulation post-hypoxia as expected (Figure 2B).

### Hypoxic Upregulation of *Stc2* Requires HIF1

**FIGURE 3:**
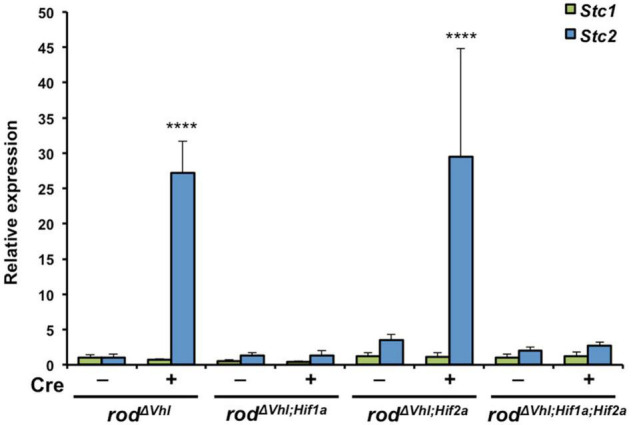
Upregulation of *Stc2* upon stabilization of HIF1A in rod photoreceptors. Relative expression of *Stc1* (green bars) and *Stc2* (blue bars) in *rod^Δ^*^Vhl^**, *rod^Δ^*^Vhl;Hif^*^1^*^a^**, *rod*^*ΔVhl;Hif*2*a*^, and *rod^Δ^^Vhl;Hif^*^1^*^a;Hif^*^2^*^a^* mice and their control lines that carried the respective floxed genes without *Opsin-cre* (–). Expression was determined by semi-quantitative real-time PCR and normalized to *Actb* levels. Shown are mean values ± SD of *N* = 3. ****P < 0.0001. Two-way ANOVA with Sidak’s multiple comparison test.

**FIGURE 4:**
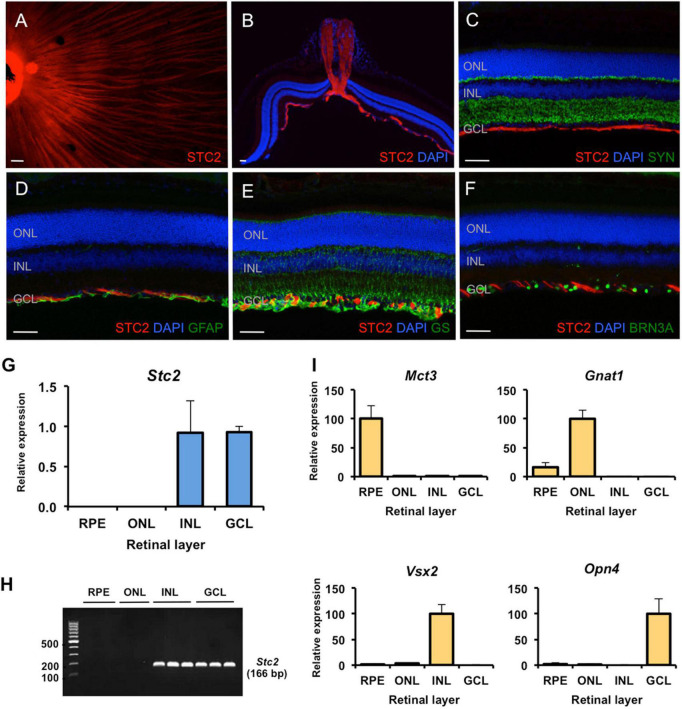
Site-specific detection of STC2 protein and mRNA expression in retinal tissue. **(A)** Immunostaining for STC2 (red) in a retinal flatmount of a 7 weeks old wild type (129S6) mouse. **(B)** Immunostaining for STC2 in a retinal section of an 11 weeks old wild type (129S6) mouse. Co-immunostainings for STC2 (red) and SYN (**C**, green), GFAP (**D**, green), GS (**E**, green), and BRN3A (**F**, green). Nuclei are stained with DAPI (blue). Scale bar: 50 μM. **(G)** Relative expression of *Stc2* mRNA in the RPE (retinal pigment epithelium), ONL (outer nuclear layer), INL (inner nuclear layer), and GCL (ganglion cell layer) that were isolated by laser capture microdissection. **(H)** The amplified PCR products from experiments shown in panel **(G)** were run on an agarose gel for visualization. *N* = 3. **(I)** Relative expression of layer-specific control genes: *Mct3* for RPE, *Gnat1* for ONL, *Vsx2* for INL, and *Opn4* for GCL. Expression was determined by semi-quantitative real-time PCR and normalized to *Actb*. Shown are mean values ± SD of *N* = 3.

To analyze the molecular requirements for hypoxic *Stc2* induction in the retina, we took advantage of several mouse lines lacking either *Vhl* (*rod^Δ^*^Vhl^**), *Vhl* and *Hif1a* (*rod^Δ^*^Vhl;Hif^*^1^*^a^**), *Vhl* and *Hif2a* (*rod^Δ^*^Vhl;Hif^*^2^*^a^**) or *Vhl*, *Hif1a* and *Hif2a* (*rod^Δ^*^Vhl;Hif^*^1^*^a;Hif^*^2^*^a^**) in rod photoreceptors (Lange et al., 2011; Barben et al., 2018). The absence of VHL activated HIF transcription factors and thus resulted in a chronic hypoxia-like response in rods. This enabled us to investigate the potential involvement of HIF1 and HIF2 transcription factors in the activation of *Stc2* in photoreceptors with an activated hypoxic response. Although our data (Figures 1B,C) suggested only a very weak or even absent (Figure 4, see below) expression of *Stc1* and *Stc2* in normal photoreceptors, activation of HIF transcription factors by means of *Vhl* deletion in rods resulted in increased retinal *Stc2* expression (Figure 3, *rod^Δ^*^Vhl^**). *Stc1*, however, was not induced in the absence of *Vhl*, resembling the results from the acute hypoxia experiment (Figure 2). *Stc2* expression was abolished in retinas of mice with the additional deletion of *Hif1a* (*rod^Δ^*^Vhl;Hif^*^1^*^a^**) or of both *Hif1a* and *Hif2a* (*rod^Δ^ *^Vhl;Hif^*^1^*^a;Hif^*^2^*^a^**) whereas the retinas of mice lacking only *Hif2a* in addition to *Vhl* (*rod^Δ^ *^Vhl;Hif^*^2^*^a^**) had *Stc2* levels comparable to those in *rod^Δ^ *^Vlhl^** mice (Figure 3). These data indicate that HIF1, not HIF2, is responsible for regulating *Stc2* in retina during hypoxic events.

### *Stc2* Is Expressed in the Inner Retina

Immunostaining on retinal flatmounts and sections of wild type mice revealed that the STC2 protein localized to axonal neurofilaments in the ganglion cell layer (GCL, Figures 4A,B). Co-stainings for markers of various retinal cell types, including SYP (synaptophysin, Figure 4C) for synapses in the plexiform layers, GFAP (glial fibrillary acidic protein, Figure 4D) for activated Müller cells and astrocytes, GS (glutamine synthetase, Figure 4E) for Müller cells and BRN3A (brain-specific homeobox protein 3A, Figure 4F) for retinal ganglion cells, suggested that STC2 localized to the neurofilament layer and did not co-localize with any of the above markers (Figures 4C–F).

Since expression of *Stc2* was not reduced in degenerated *rd10* retinas lacking all photoreceptors, *Stc2* may not normally be expressed in rods (see above). To elucidate the retinal layers expressing *Stc2* in more detail, we separated the retinal pigment epithelium (RPE), outer nuclear layer (ONL), inner nuclear layer (INL), and the GCL by laser capture microdissection and analyzed *Stc2* expression by real-time PCR. Results showed that *Stc2* was strongly and exclusively expressed in the INL and GCL, but not in the ONL or RPE of the normal retina (Figures 4G–I). Layer specific markers for the RPE (*Mct3*, monocarboxylate transporter 3), ONL (*Gnat1*, guanine nucleotide-binding protein G subunit alpha-1, for rods), INL (*Vsx2*, visual system homeobox 2, for bipolar cells), and GCL (*Opn4*, melanopsin, for ganglion cells) verified the separation of the individual retinal layers (Figure 4I).

### Both *Stc1^–/–^* and *Stc2^–/–^* Mice Have a Normal Retinal Morphology

**FIGURE 5:**
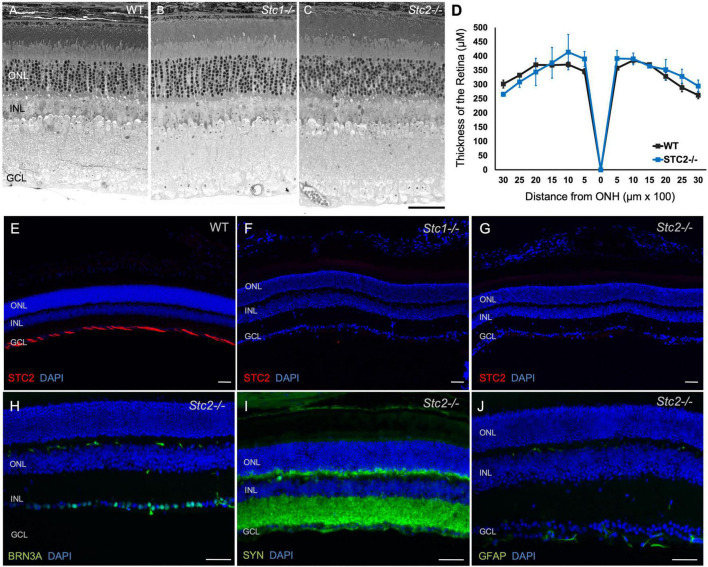
Retinal morphology in the absence of STC1 and STC2. Retinal morphology of wild type (WT, C57BL/6) **(A)**, *Stc1^–/–^* **(B)** and *Stc2^–/–^*, **(C)** mice. **(D)** Spidergram of the retinal thickness of WT (C57BL/6, black line) and *Stc2^–/–^* (blue line) mice; *N* = 3, ONH: Optic Nerve Head Immunostainings for STC2 (red) in retinal sections of wild type (C57BL/6) **(E)**, *Stc1^–/–^* **(F)**, and *Stc2^–/–^* **(G)** mice. Immunostainings for BRN3A **(H)**, SYN **(I)**, and GFAP **(J)** in retinal sections of *Stc2^–/–^* mice. DAPI (blue) was used as nuclear stain. Images were acquired at 200 μm from the optic nerve head. Mice were 6 months (wild type) or 9 months (*Stc1^–/–^* and *Stc2^–/–^*) of age. Shown are representative images of *N* = 3 retinas per genotype. Scale bar: 50 μm.

Two *Stc1* knockout mouse lines display a dwarf phenotype (Filvaroff et al., 2002; Varghese et al., 2002) while a third line was reported without a significant phenotype (Chang et al., 2005). Transgenic mice overexpressing human STC2 are about 45% smaller than their littermates (Gagliardi et al., 2005) while *Stc2^–/–^* mice are 10–15% larger and grow faster than wild type mice (Chang et al., 2008). Although not completely congruent, published data thus indicate a potential involvement of stanniocalcins in development (Filvaroff et al., 2002; Varghese et al., 2002). To test a potential influence of stanniocalcins on retinal development and/or maintenance we investigated the retinal morphology in eyes of 9 months old *Stc1^–/–^* and *Stc2^–/–^* mice. Surprisingly, morphology and retinal architecture of both knockout mice were indistinguishable from wild type mice (Figures 5A–C). This was further supported by the similar retinal thickness of wild type and *Stc2^–/–^* mice (Figure 5D). Immunolabeling of STC2 in wild type mice was detected in the NFL and was absent in the *Stc2^–/–^* mice as expected, but interestingly, was also not detectable in *Stc1^–/–^* mice (Figures 5E–G). Immunolabeling for BRN3A, SYN and GFAP in *Stc2^–/–^* mice showed normal expression patterns (Figures 5H–J).

## Discussion

Neuroprotection slows the progression of retinal degeneration and extends the therapeutic window for additional treatments. Hence there is an active pursuit for factors that can protect retinal cells. Protective factors are often involved in pro-survival signaling pathways that are activated when the tissue experiences stress. Such a stress can be caused by reduced oxygen availability leading to local hypoxia that has been implicated in retinal degeneration and disease (Lange and Bainbridge, 2012). Since several studies have implicated both secreted glycoproteins STC1 and STC2 in the response to hypoxia (Chang et al., 2003; Joshi, 2020), we investigated expression and hypoxic regulation of these two genes in the retina. Surprisingly, and in contrast to other tissues (Yeung et al., 2005; Westberg et al., 2007a,b), only *Stc2* and not *Stc1* was responsive to hypoxia in the retina (Figure 2). Two consensus HIF1 binding sites were recognized and characterized in the promoter region of the *Stc2* gene in previous *in vitro* studies (Law and Wong, 2010a). In line with this finding, we showed that upregulation of *Stc2* expression in *rod^Δ^ *^Vhl^** mice depended on the transcription factor HIF1A (Figure 3). A search for hypoxia-response elements in the promoter of *Stc1* did not yield conclusive results but suggested that *Stc1* was inhibited by factor inhibiting HIF (FIH) (Law et al., 2010). However, unless *Stc1* and *Stc2* are expressed in separate cell types, it seems unlikely that FIH accounts for the lack of *Stc1* induction in hypoxia in the retina as its action would affect HIF1 activity in general and thus expression of *Stc2* as well.

Our results showed that *Stc1* and *Stc2* were highly expressed in the retina of wild type mice and their expression was largely unaffected by the loss of photoreceptors during retinal degeneration (Figure 1). This suggests that the two stanniocalcin genes are mainly expressed in cells of the inner retina of wild type mice, a conclusion supported by our laser capture microdissection data (Figure 4). The localization of STC2 protein on neurofilaments further strengthens this conclusion. In the *rod^Δ^ *^Vhl^** mice that serve as a model for an active hypoxic response, *Stc2* gene is also expressed in photoreceptors where HIF1 is stabilized (Figure 3). However, the protein is still localized in the neurofilaments, and to some extent in Muller cells. STCs are secreted glycoproteins and we hypothesize that they may be produced by cells of the INL and GCL in the normal retina, or in response to hypoxia in other cell types, and secreted and transported to the NFL where they are required. The exact mechanism of this secretion, transport and their function in the NFL is not clear and merits further investigation.

The function of stanniocalcins in the retina is unknown but the surprising lack of STC2 protein in *Stc1^–/–^* mice hints at a potential cross-talk in the regulation of the two stanniocalcins (Figure 5). Following this intriguing result, it would have been interesting to know if STC1 is missing from Stc2–/– mice as well. However, our primary goal was to analyze hypoxia-regulated genes in the retina, and since the expression level of *Stc1* was low and not regulated by hypoxia we did not further test STC1 protein levels. Nevertheless, stanniocalcins may not have essential functions for retinal development and maintenance as the retinal morphologies of *Stc1^–/–^* and *Stc2^–/–^* mice appeared normal (Figure 5) despite reported effects of the knockouts on general animal size (Filvaroff et al., 2002; Varghese et al., 2002; Chang et al., 2005, 2008). It is possible that lack of STC1 and/or STC2 does not affect the retina under normal physiological conditions, but that the two stanniocalcins are needed under stress conditions. Hence, it would be interesting to investigate how *Stc1^–/–^* and *Stc2^–/–^* mice respond when subjected to hypoxic stress or to photoreceptor loss in models of retinal degeneration.

Several publications report protective activities of STC1 in the retina including a study concluding that intravitreal injections of STC1 rescued degeneration in S334ter-3 and RCS rats, which are models for fast and slow, respectively, photoreceptor degeneration (Roddy et al., 2012). It would be of interest to investigate whether maintaining high retinal levels of STC1 prevents or slows the progression of degeneration in the *rd10* mouse, as it was reported for rats (Roddy et al., 2012). Since *Stc2* showed a strongly elevated expression during hypoxic stress, it may be equally important to test whether high levels of STC2 would be neuroprotective in the retina, similar to STC1 in other tissues. Such data may make stanniocalcins attractive targets for the development of potential therapeutic strategies.

Taken together our study investigates for the first time the expression of stanniocalcins in the normal, degenerating and hypoxic retina. The differential regulation of *Stc1* and *Stc2* under hypoxic conditions indicates the possibility of distinct and non-redundant functions for the two proteins in the retina. We show HIF1A-mediated regulation of *Stc2* in photoreceptors lacking *Vhl*, which merits further study of the potential of STC2 as a retinal neuroprotective candidate.

## Data Availability Statement

The original contributions presented in this study are included in the article/supplementary material, further inquiries can be directed to the corresponding author.

## Ethics Statement

The animal study was reviewed and approved by the Veterinary authority of Zurich, Switzerland.

## Author Contributions

CG and DA: conceptualization and analysis. DA, MS, and JK: experiments. AC and RR: STC KO mice related experiments. DA: writing – original draft. DA, MS, RR, and CG: writing – revision and editing. CG: project administration and funding acquisition. All authors contributed to the article and approved the submitted version.

## Conflict of Interest

The authors declare that the research was conducted in the absence of any commercial or financial relationships that could be construed as a potential conflict of interest.

## Publisher’s Note

All claims expressed in this article are solely those of the authors and do not necessarily represent those of their affiliated organizations, or those of the publisher, the editors and the reviewers. Any product that may be evaluated in this article, or claim that may be made by its manufacturer, is not guaranteed or endorsed by the publisher.
